# Small RNA SmsR1 modulates acidogenicity and cariogenic virulence by affecting protein acetylation in *Streptococcus mutans*

**DOI:** 10.1371/journal.ppat.1012147

**Published:** 2024-04-15

**Authors:** Jing Li, Qizhao Ma, Jun Huang, Yaqi Liu, Jing Zhou, Shuxing Yu, Qiong Zhang, Yongwang Lin, Lingyun Wang, Jing Zou, Yuqing Li

**Affiliations:** 1 State Key Laboratory of Oral Diseases, National Clinical Research Center for Oral Diseases, West China Hospital of Stomatology, Sichuan University, Chengdu, China; 2 Department of Pediatric Dentistry, West China Hospital of Stomatology, Sichuan University, Chengdu, China; 3 Department of Operative Dentistry and Endodontics, West China Hospital of Stomatology, Sichuan University, Chengdu, China; 4 Section of Infectious Diseases, Department of Internal Medicine, Yale University School of Medicine, New Haven, Connecticut, United States of America; National Jewish Health, UNITED STATES

## Abstract

Post-transcriptional regulation by small RNAs and post-translational modifications (PTM) such as lysine acetylation play fundamental roles in physiological circuits, offering rapid responses to environmental signals with low energy consumption. Yet, the interplay between these regulatory systems remains underexplored. Here, we unveil the cross-talk between sRNAs and lysine acetylation in *Streptococcus mutans*, a primary cariogenic pathogen known for its potent acidogenic virulence. Through systematic overexpression of sRNAs in *S*. *mutans*, we identified sRNA SmsR1 as a critical player in modulating acidogenicity, a key cariogenic virulence feature in *S*. *mutans*. Furthermore, combined with the analysis of predicted target mRNA and transcriptome results, potential target genes were identified and experimentally verified. A direct interaction between SmsR1 and 5’-UTR region of *pdhC* gene was determined by *in vitro* binding assays. Importantly, we found that overexpression of SmsR1 reduced the expression of *pdhC* mRNA and increased the intracellular concentration of acetyl-CoA, resulting in global changes in protein acetylation levels. This was verified by acetyl-proteomics in *S*. *mutans*, along with an increase in acetylation level and decreased activity of LDH. Our study unravels a novel regulatory paradigm where sRNA bridges post-transcriptional regulation with post-translational modification, underscoring bacterial adeptness in fine-tuning responses to environmental stress.

## Author summary

Post-transcriptional regulation and post-translational modification are widely present in physiological circuits due to their low energy consumption and rapid response to the environment. sRNA is an important post-transcriptional regulatory factor in bacterial response to environmental stress, and bacteria also use sRNA as a key signal to control multicellular behaviors such as biofilm formation and quorum sensing to regulate virulence. *S*. *mutans* is an intractable cariogenic pathogen, but the role of sRNA in *S*. *mutans* is poorly understood. Here, we elucidated the virulence regulation and mechanism of sRNA SmsR1 in *S*. *mutans* by means of transcriptomics, acetyl-proteomics and animal models, and found its relationship with protein acetylation (post-translational modification), and a new role of sRNA physiological regulatory network was shown. This study presents an innovative perspective that a sRNA in *S*. *mutans* is involved in the correlation between post-transcriptional regulation and post-translational modification.

## Introduction

Microorganisms maintain life processes and regulate metabolism through dynamic and intricate regulatory networks, ensuring their survival in complex and changeable environments. Key to this adaptability is their use of different regulatory mechanisms operating at the gene, transcript, and protein levels [1]. These regulatory processes encompass second messengers [2], transcription factors at the transcriptional level, post-transcriptional control by small non-coding RNA (sRNA) [3,4], protein expression at the translational level, and finally post-translational modifications (PTM) such as acetylation and phosphorylation [5]. In the multifaceted life activities of bacteria, these regulatory factors are not isolated but rather intricately interlocked, coordinating different aspects of growth and metabolism, including biofilm formation, antibiotic resistance, virulence, and adaptability to the environment. Among these factors, sRNA and PTM have emerged as pivotal regulatory mechanisms that enable bacteria to sense and respond to environmental signals with minimal energy expenditure and remarkable swiftness [6,7].

sRNAs, a class of RNA molecules typically spanning 50–500 nucleotides, characterized by intrinsic Rho-independent terminator in the secondary structure with stabilizing stem-loop, primarily function by engaging in limited or imperfect base-pairing via complementarity with target mRNA [8]. By binding near the ribosome binding site (RBS) of their target mRNA, sRNAs can effectively impede translation, while in other cases may employ diverse mechanisms such as altering ribosome binding or target mRNA stability, to fine-tune gene expression [9,10]. Furthermore, certain sRNAs such as CsrB and 6S RNA in *E*. *coli* transcend traditional base-pairing rules to directly bind to a specific protein and modulate its function [11–13]. The diverse mechanisms of action of sRNAs make them key potent regulators in extensive physiological circuits, including stress response (e.g., temperature, pH, oxidative stress, and osmotic stress), quorum sensing, biofilm formation and virulence [6,14–16]. The lightweight and streamlined nature of sRNAs circumventing the translation step offers an energy-saving alternative to protein-based transcription factors (TFs) making them advantageous for rapid responses to external stimuli.

In intricate regulatory networks, sRNAs do not function in isolation but rather display a remarkable propensity to cross-talk, not only with an array of genes but also with second messengers or TFs, making the role of sRNAs central in regulatory networks [17]. For example, the *Yersinia pestis* sRNA HmsB enhances production of the second messenger Cyclic di-GMP (c-di-GMP) [18]. sRNAs can control the transcription factor *rpoE* at the transcriptional level to mediate the regulation of envelope stress response [19]. Despite these established roles, there remains a paucity of studies elucidating the relationship between sRNAs and PTMs.

Similar to post-transcriptional regulation, bacteria also excel in their ability to rapidly adapt to variable environments and modulating virulence through PTM mechanisms [20]. By introducing covalent modifications to specific amino acid residues, PTMs expand the functional landscape of proteins. Such modifications can redefine the functionality of proteins by altering their charge, conformation, and interactions [21]. Among the myriads of PTMs, lysine acetylation, facilitated by acetyl donors like acetyl coenzyme A (Ac-CoA) and acetyl phosphate (AcP), has surged to prominence due to it ubiquitous nature and profound implications in bacterial virulence and metabolism [22–24]. For example, the link between acetylation and virulence in *Salmonella* is substantiated by studying acetyltransferase Pat deletion mutants, which exhibit reduced expression of SPI-1 and decreased cell invasion capabilities [25].

*Streptococcus mutans* is the primary bacterium causing dental caries, which is the most prevalent chronic oral infectious disease globally [26–28]. Due to its strong ability to form biofilms and generate a rich extracellular matrix, it can firmly adhere to the tooth surface for colonization. Its robust acid-producing ability promotes the gradual demineralization and disintegration of the tooth surface, leading to the formation of caries cavities. Furthermore, its resistance to acidic environmental pressures enables it to survive in the complex and varied oral environment.

Generally, sRNAs lack the ability to encode proteins. Recently, it has been discovered that some sRNAs have dual functions, acting both as base-pairing RNA and as peptide-coding mRNA [29]. In *S*. *mutans*, several sRNAs have been experimentally identified [30], in which sRNA SmsR1 could potentially exhibit dual functions. It contains the coding sequence for ComS (a 17-amino acid peptide), which is part of the quorum sensing system ComRS [31]. Previous studies have found that ComS acts as a precursor of signal peptide XIP, regulating intercellular communication and bacteriocin production in *S*. *mutans* [32,33]. However, the mechanism of sRNA SmsR1 acts as a base-pairing RNA in regulating *S*. *mutans* physiology and virulence is still unknown. Our current investigations unveil the crosstalk between the sRNA SmsR1 and lysine acetylation in *S*. *mutans* and shed light on their roles in acidogenicity and adaption to acidic stress. Beyond its post-transcriptional regulation, SmsR1 indirectly regulates lactate dehydrogenase (LDH) activity without altering its mRNA expression levels. Furthermore, we verified that *pdhC* is the potential target gene of SmsR1 through target mRNA prediction, transcriptome analysis, and *in vitro* binding experiments. Overexpression of SmsR1 elevated the intracellular acetyl-CoA levels and protein acetylation profile. Mass spectrometry and western blotting assays revealed that the acetylation level of LDH increased significantly upon SmsR1 overexpression, causing a decrease in LDH enzymatic activity. Taken together, our study unveils the crosstalk between sRNA and lysine acetylation, offering insights into the broader regulatory mechanisms that bacteria utilize to fine-tune their physiological responses to diverse environmental signals.

## Results

### SmsR1 affects the acidogenicity of *S*. *mutans*

**Fig 1:**
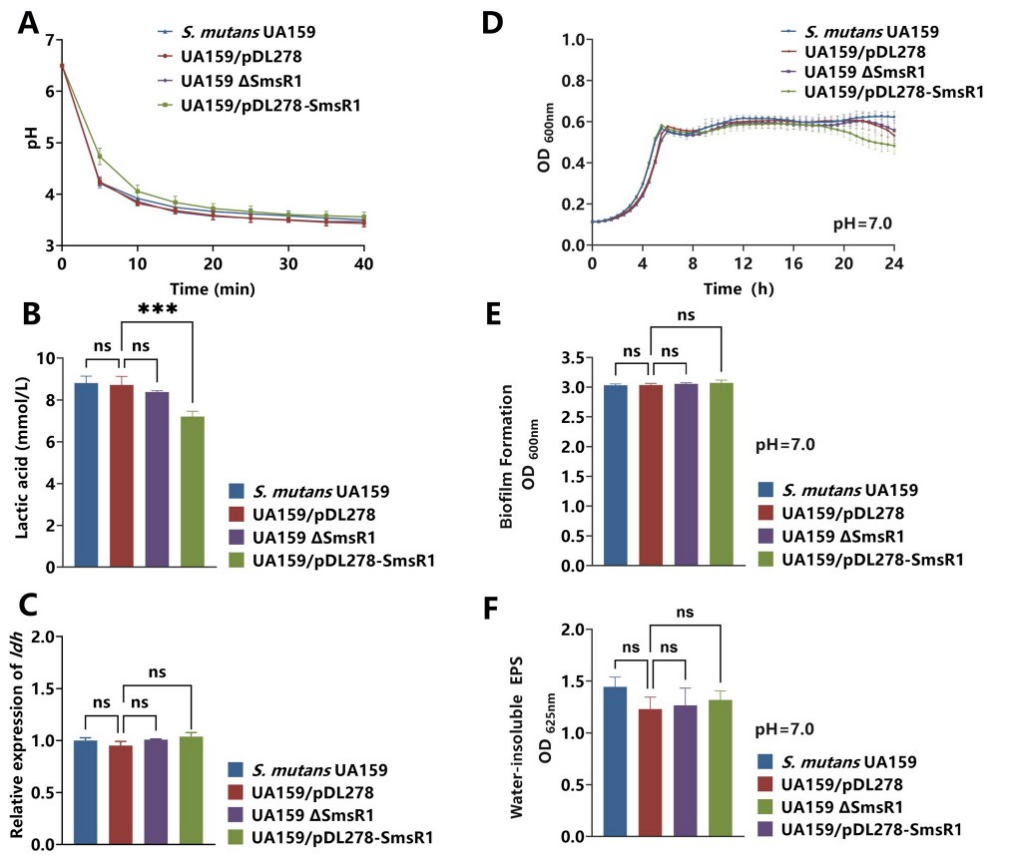
Effects on growth and virulence phenotypes of *SmsR1* overexpression *in vitro*. (A) pH values of culture supernatants of the strains treated with 1% glucose for 40 minutes. (B) The lactic acid production as measured by a lactic acid assay kit. (C) The mRNA levels of *ldh* as determined by qRT-PCR using the 2 ^-ΔΔCT^ method. (D) Growth curves of *S*. *mutans* UA159, UA159/pDL278 (empty vector) or UA159/pDL278-SmsR1 (overexpression) in BHI broth (pH = 7) for 24 hours. After the strains were cultured under anaerobic conditions in 1% BHI-s broth (pH = 7.0) for 24 hours, biofilm biomass as measured by crystal violet staining (E), and water-insoluble exopolysaccharides as measured by sulfuric anthrone reaction (F). Results are expressed as mean and standard deviation (SD), n = 3 biological replicates, statistical significance was determined using one-way analysis of variance (ANOVA) and Tukey’s test were performed to compare data between multiple groups; *P* < 0.05 was considered statistically significant. (ns, *P* > 0.05; *, *P* <0.05; **, *P* < 0.01; ***, *P* <0.001; ****, *P* <0.0001).

Based on global sequencing and northern blot verification of sRNA in *S*. *mutans* by Krieger MC et al [30], and considering that sRNA is mostly produced in response to environmental stress. [34]. especially when virulence-related phenotypes are involved, overexpression may be a an acceptable approach to screen for sRNA functions [35].We constructed 10 overexpression strains of verified sRNAs in *S*. *mutans*, and included the empty vector as a control. Glycolytic pH drop assays were performed to evaluate the acidogenicity phenotype, which is the direct cariogenic virulence of *S*. *mutans* (S1 Fig). We found that SmsR1 overexpression weakened the acid production rate of *S*. *mutans* in the presence of 1% glucose (Fig 1A). Since the main acid-producing product of *S*. *mutans* glycolysis is lactic acid, we tested lactic acid production in these strains. We found that lactic acid accumulation in the SmsR1 overexpressing strain was less than that in the control, indicating that the lactic acid production catalyzed by lactate dehydrogenase (LDH) was reduced (Fig 1B). We speculated that the gene expression level of *ldh* was regulated by SmsR1. Interestingly, we found that the mRNA levels of *ldh* in different strains were not significantly down-regulated or up-regulated (Fig 1C). Therefore, *ldh* mRNA may not be the direct target of SmsR1 through complementary base pairing regulation. It is possible that the protein levels or enzymatic activity of LDH were affected.

### SmsR1 does not affect the growth and biofilm formation in *S*. *mutans*

To evaluate the effect of SmsR1 on the growth of *S*. *mutans* we tested the growth kinetics in BHI broth and found that overexpression of SmsR1 was not significantly different from the control (Fig 1D). After biofilm formation, *S*. *mutans* can rapidly create an acidic environment locally. The biomass and EPS quantity of the biofilm are key factors influencing acid production under biofilm conditions. As shown in Fig 1E and 1F, biofilm formation and production of water-insoluble exopolysaccharides (EPS) did not change significantly upon SmsR1 overexpression (*P* > 0.05). These results suggested that the reduction in lactic acid producing capacity upon SmsR1 overexpression was not due to changes in growth kinetics or biofilm formation.

### The impact of SmsR1 on the acidogenicity of *S*. *mutans* is not related to ComS

**Fig 2:**
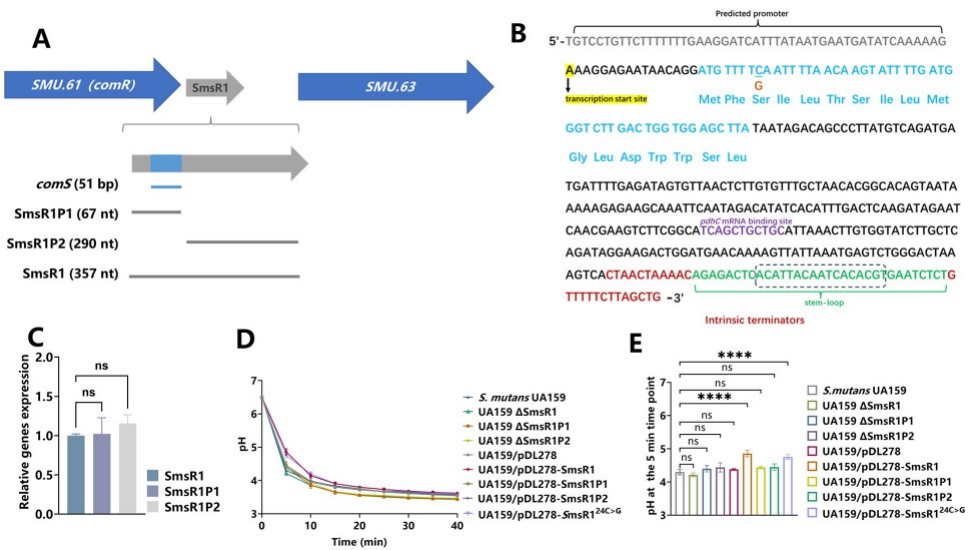
Characterization of SmsR1 in *S*. *mutans*. (A) The position of SmsR1 in the *S*. *mutans* UA159 genome is shown. (B) Sequence of SmsR1. Transcription start sites are highlighted in yellow, coding region of ComS is denoted in blue text, purple text denotes the region of SmsR1 that base pairs with the target mRNA *pdhC*. Intrinsic terminator was predicted by ARNold Web Server and shown in red, the predicted stem-loop structure is represented in green, and the part enclosed by the box forms the loop. (C) The expression of SmsR1, SmsR1P1, SmsR1P2 in *S*. *mutans* UA159 was determined by qRT-PCR using the 2 ^-ΔΔCT^ method. (D) pH values of culture supernatants of strains treated with 1% glucose for 40 minutes. (E) pH values of culture supernatants of strains treated with 1% glucose at the 5 min time point. Results are expressed as mean and SD; n = 3 biological replicates, statistical significance was determined using one-way analysis of variance (ANOVA) and Tukey’s test were performed to compare data between multiple groups; *P* < 0.05 was considered statistically significant. (ns, *P* > 0.05; *, *P* <0.05; **, *P* < 0.01; ***, *P* <0.001; ****, *P* <0.0001).

SmsR1 is 357 nt in length and located in the intergenic region between *comR* and *SMU*.*63* (Fig 2A). The sequence encoding ComS was located in the front part of it, therefore, we named it SmsR1P1 and the remaining part is called SmsR1P2 to distinguish them. The sequence information of SmsR1 is shown in Fig 2B. It has a predicted promoter and an internal Rho-independent terminator.

To confirm whether SmsR1P1 and SmsR1P2 are co-transcribed as a single unit when cultured in BHI broth, we performed qRT-PCR to detect the segmented sequences. The results proved that the CT values and transcription levels were consistent (Fig 2C). Since *ComS* is located within SmsR1, we wondered whether the reduced acid production phenotype upon SmsR1 overexpression was due to ComS. We constructed overexpression and deletion mutants of the anterior and posterior segments of SmsR1, named UA159/pDL278-SmsR1P1, UA159/pDL278-SmsR1P2, UA159 ΔSmsR1P1, and UA159 ΔSmsR1P2. Surprising, these mutants did not yield any significant differences in acid production rate when compared to the control group (Fig 2D and 2E). This confused us about the structural integrity affecting the function of sRNA. In Storz G’s study of the dual-function RNA AzuCR, they mutated the third codon into a stop codon to investigate AzuCR’s function as an sRNA. We were inspired by this approach [36,37]. Based on their approach, we mutated the C at site 24 (underlined in Fig 2B) into G (orange in Fig 2B) to introduce the TGA stop codon (SmsR1^24C>G^). In this strain, *ComS* is not translated normally, thus eliminating the role of *ComS* overexpression while simultaneously ensuring the structural integrity SmsR1. We observed the reduced acid production phenotype in a strain expressing SmsR1^24C>G^ (Fig 2D), further demonstrating that SmsR1 acted as a sRNA to regulate the acidogenicity phenotype of *S*. *mutans*. To avoid the influence of possible overexpression of *ComS* on the results, we used SmsR1^24C>G^ as a reference group in subsequent experiments where needed.

### Overexpression of SmsR1 affects the survival of *S*. *mutans* in the acidic environment

**Fig 3:**
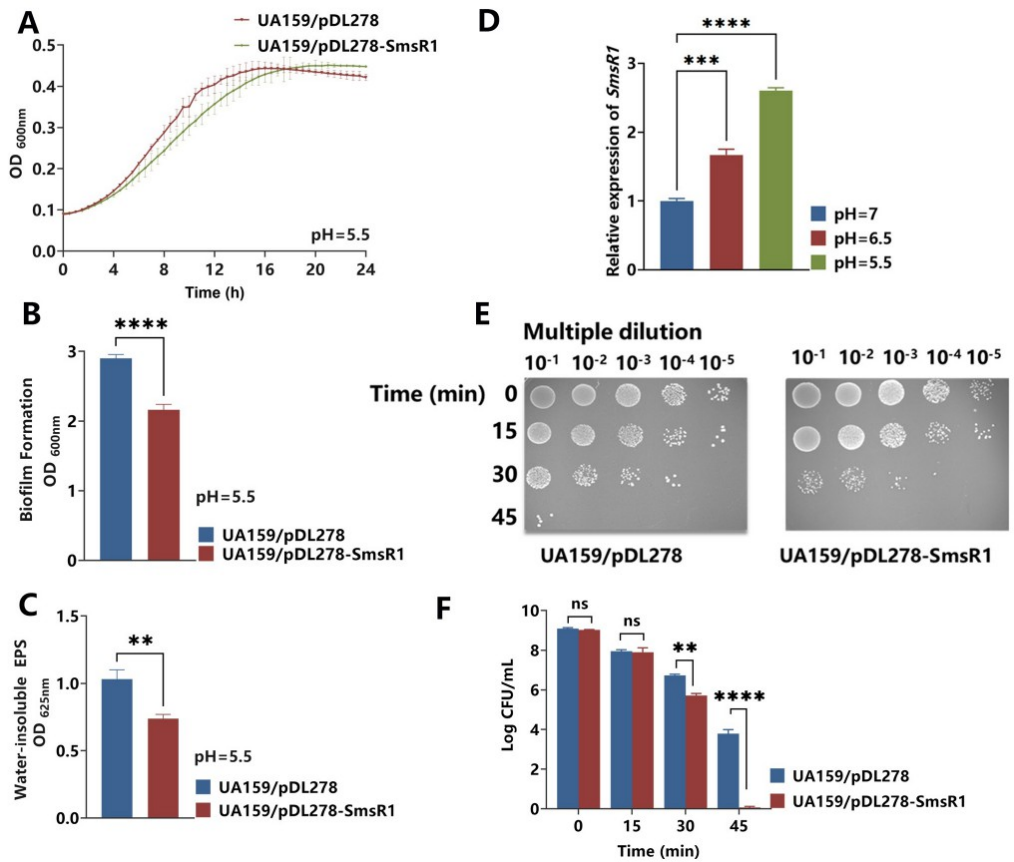
Impact on ATR of UA159/pDL278-SmsR1. (A) Growth curves of the strains grown in BHI broth (pH = 5.5) for 24 hours. After the strains were cultured under anaerobic conditions in 1% BHI-s broth (pH = 5.5) for 24 hours, biofilm biomass as measured by crystal violet staining (B), and water-insoluble exopolysaccharides as measured by sulfuric anthrone reaction (C). (D) After treating *S*. *mutans* UA159 under different pH conditions (pH = 7, 6.5 and 5.5), the levels of SmsR1 transcript were determined by qRT-PCR using the 2 -^ΔΔCT^ method. (E) Survival of these strains after acid killing with glycine at pH = 2.8 for 0, 15, 30 and 45 minutes. (F) CFU numbers of surviving cells in strains after acid killing treatment. Results are expressed as mean and SD; n = 3 biological replicates, statistical significance was determined using one-way analysis of variance (ANOVA) and Tukey’s test were performed to compare data between multiple groups; *P* < 0.05 was considered statistically significant. (ns, *P* > 0.05; *, *P* <0.05; **, *P* < 0.01; ***, *P* <0.001; ****, *P* <0.0001).

Acid tolerance as another core factor in the cariogenic virulence can help *S*. *mutans* produce acidic substances to demineralize the tooth surface and ensure better survival under acid stress. We verified the acid tolerance abilities of different *S*. *mutans* strains by performing the acid tolerance response (ATR) experiments. First, the growth kinetics of the control and SmsR1 overexpressing strains were determined in BHI broth at pH 5.5 (Fig 3A). In contrast to growth in BHI broth at pH 7 (Fig 1C), SmsR1 overexpressing strain showed a growth lag phenotype. At the same time, the amount of biofilm and water-insoluble EPS of UA159/pDL278-SmsR1 were also significantly reduced (Fig 3B and 3C), suggesting that its growth was more compromised in an acidic environment. We determined SmsR1 expression levels of the parent strain treated for 30 minutes at different pH conditions (pH = 7, 6.5 and 5.5), and found that the expression of SmsR1 increased significantly with the harsher acidic environment (Fig 3D). This result is consistent with those reported by Krieger, M. C *et al* [30], which showed that acid stress induced the expression of SmsR1. Simultaneously, we used pH = 2.8 glycine to simulate acid-killing conditions (Fig 3E and 3F) and found that the survival rate of the SmsR1 overexpressing strain reduced with increasing time of acid threat (30 min, 45 min).

### RNA-seq analysis and bioinformatics prediction reveal potential targets of SmsR1

**Fig 4:**
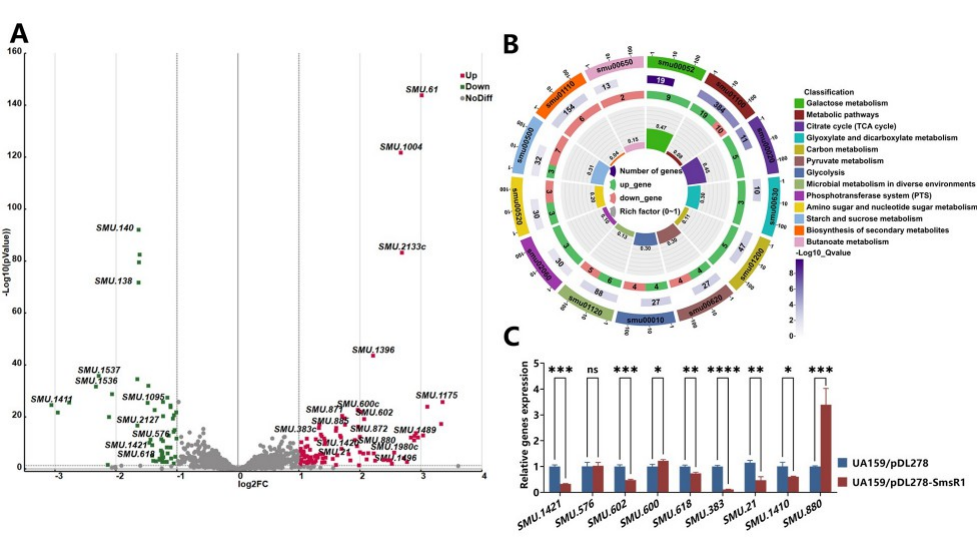
Detecting potential SmsR1 target mRNAs based on transcriptome results. (A) Volcano plot shows the differences in gene expression between UA159/pDL278 and UA159/pDL278-SmsR1. Upregulated genes with statistically significant differences (*P* < 0.05) are shown in red, while downregulated genes are shown in green. (B) The KEGG pathway enrichment analysis of DEGs in UA159/pDL278-SmsR1. Upregulated genes are shown in red and downregulated genes are shown in green. KEGG, Kyoto Encyclopedia of Genes and Genomes. (C) The mRNA levels of potential target genes as determined by qRT-PCR using the 2 -^ΔΔCT^ method. Results are expressed as mean and SD; n = 3 biological replicates, statistical significance was determined using one-way analysis of variance (ANOVA) and Tukey’s test were performed to compare data between multiple groups; *P* < 0.05 was considered statistically significant. (ns, *P* > 0.05; *, *P* <0.05; **, *P* < 0.01; ***, *P* <0.001; ****, *P* <0.0001).

To determine the possible mechanism of SmsR1 regulation of *S*. *mutans*, we conducted transcriptional sequencing to compare the SmsR1 overexpressing strain with the control, and further predicted potential mRNA targets of SmsR1 using RNA-RNA interaction prediction tool *IntaRNA* (uni-freiburg.de) [38]. The results showed significant changes in the expression of 147 genes in the transcriptome data of the SmsR1 overexpressing strain (S1 Table), of which 92 genes were up-regulated and 55 were down-regulated (Fig 4A). We used KEGG analysis for pathway enrichment and found that galactose metabolism, citrate pyruvate cycle, and pyruvate metabolism were significantly changed (Fig 4B). We predicted the top 100 scoring target mRNAs of SmsR1 in *S*. *mutans* UA159 using *IntaRNA* (S2 Table). We carried out qRT-PCR assays to verify the transcriptome results. The expression levels of *SMU*.*1421* (pyruvate dehydrogenase E2 component, *pdhC*), *SMU*.*1410* (putative reductase), *SMU*.*880* (multiple sugar-binding ABC transporter, *msmG*), and *SMU*.*618* were consistent with the transcription results (Fig 4C).

### SmsR1 overexpression elevates acetyl-CoA levels in *S*. *mutans*, increases LDH acetylation, and decreases LDH activity

**Fig 5:**
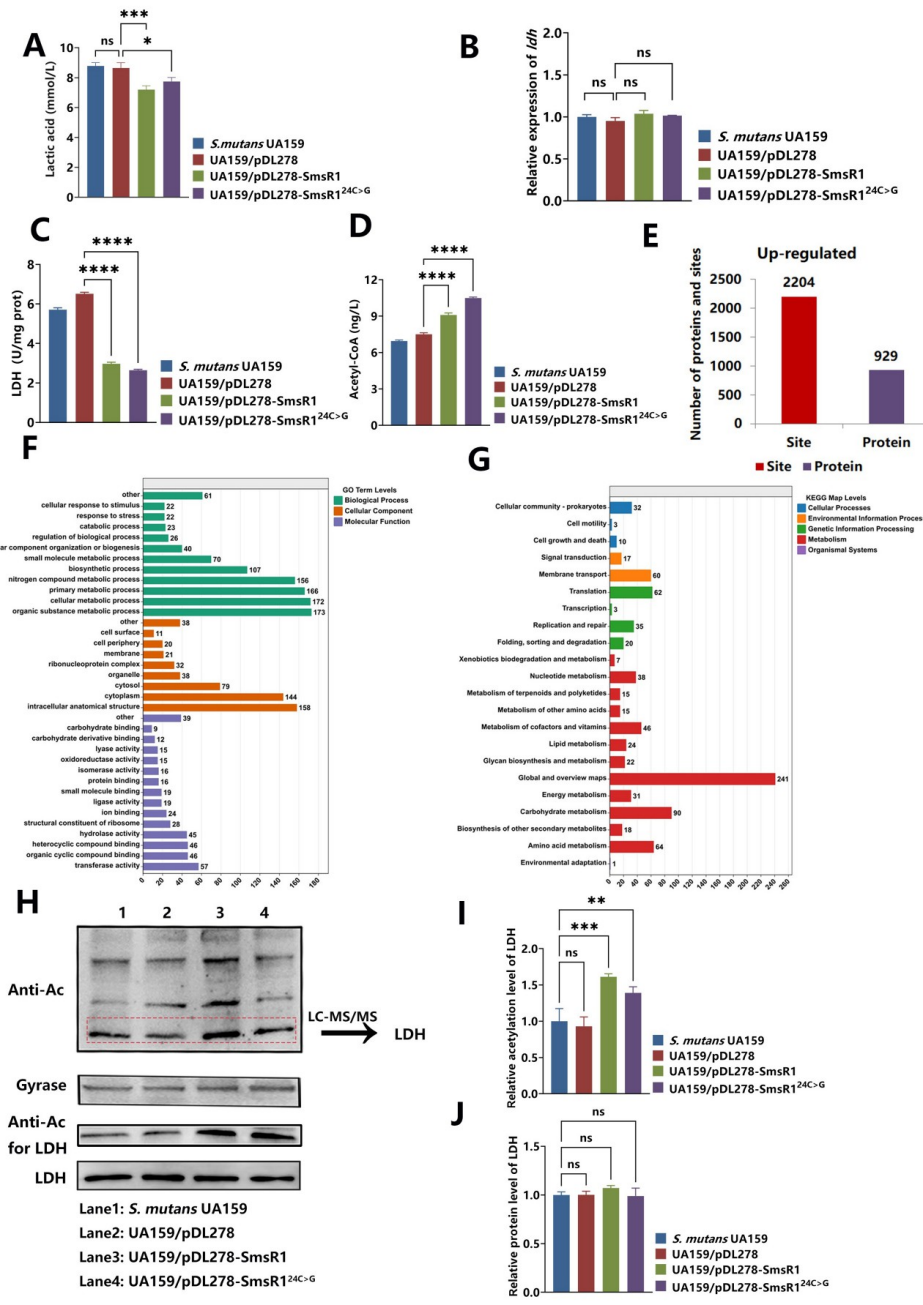
Effect of SmsR1 overexpression on lactic acid production, LDH activity, mRNA level and acetylation level. (A) The lactic acid production of the strains. (B) The mRNA levels of *ldh* as determined by qRT-PCR using the 2 -^ΔΔCT^ method. (C) The LDH enzymatic activity levels of the strains as evaluated by the LDH activity kit. (D) The level of Ac-CoA of the strains as calculated by ELISA assay. (E) The amounts of upregulated acetylated proteins and sites in an SmsR1 overexpressing strain compared with the control group UA159/pDL278 at pH 7 conditions. (F) GO analysis of upregulated acetylated proteins upon SmsR1 overexpression. (G) KEGG pathway enrichment analysis of upregulated acetylated proteins in the SmsR1 overexpressing strain. (H) Total extracted proteins of strains as analyzed by anti-acetyl lysine western blotting. (I) Anti-acetyl lysine of LDH bands were quantified with ImageJ software and were normalized to the control group. (J) Anti-LDH bands were quantified with ImageJ software and were normalized to the control group. Results are expressed as mean and SD; n = 3 biological replicates, statistical significance was determined using one-way analysis of variance (ANOVA) and Tukey’s test were performed to compare data between multiple groups; *P* < 0.05 was considered statistically significant. (ns, *P* > 0.05; *, *P* <0.05; **, *P* < 0.01; ***, *P* <0.001; ****, *P* <0.0001).

In the above results, we found that the acidogenicity of the SmsR1 overexpressing strain was weakened, and lactic acid production was reduced, but the expression level of *ldh* was not significantly changed (Fig 5A and 5B). Further, analysis of the enzymatic activity of LDH showed that strains overexpressing SmsR1 and SmsR1^24C>G^ displayed significantly lower activity than that the control group (Fig 5C). The above experiments confirmed that the decreased acid-producing ability of the above two strains was caused by the decrease of LDH enzymatic activity.

In our previous study [39] we found that the enzymatic activity of LDH was affected by acetylation modification. Therefore, we first detected changes in the amount of acetyl-CoA (Ac-CoA), the main donor of acetylation, to determine whether acetylation might be regulated upon SmsR1 overexpression. We extracted the total protein of the strains in the logarithmic growth phase and assessed the amount of Ac-CoA by ELISA assays. Ac-CoA levels of strains overexpressing SmsR1 and SmsR1^24C>G^ were significantly higher than that of the control (Fig 5D). Acetyl-proteomics was used to further confirm whether the increase in Ac-CoA affected the overall acetylation level upon SmsR1 overexpression. As a result, we identified a total of 2204 upregulated acetylated sites in 929 proteins by acetyl-proteomics (Fig 5E and S3 Table). To better understand the biological significance of acetylation, we further performed Gene Ontology (GO) and Kyoto Encyclopedia of Genes and Genomes (KEGG) pathway analyses of acetylated proteins. GO analysis of this acetylome showed that acetylated proteins were primarily related to metabolic process and predominantly occurred in the cytosol with diverse binding activities (Fig 5F). The KEGG pathway analysis revealed that acetylated proteins were enriched in metabolic pathways, particularly carbohydrate metabolism (Fig 5G). Notably, further analysis of acetylated proteins in metabolic pathways revealed that the terminal enzyme lactate dehydrogenase (LDH), responsible for lactate production in glycolysis in *S*. *mutans* is acetylated. These results were consistent with the observed decreased acidogenicity in upon SmsR1 overexpression.

To ensure that the total protein content of each group is consistent (S2 Fig), we also profiled protein acetylation levels in the control and SmsR1 overexpressing strains by Western blotting. As shown in Fig 5H, the total protein acetylation level of the overexpressing strain was indeed significantly different from that of the control. The total protein acetylation level upon SmsR1 overexpression was relatively increased and there were prominent bands at 37 kDa in the lanes corresponding to SmsR1 and SmsR1^24C>G^ overexpression (Fig 5H). We then analyzed the protein bands with significantly increased acetylation modification by LC-MS/MS. The mass spectrum results identified this band as LDH and confirmed that SmsR1 overexpression affected the Ac-CoA distribution balance (S4 Table and S3 Fig). Ac-CoA is involved in the physiological activities of *S*. *mutans* that leads to increasing acetylation levels of LDH (Fig 5I) and reduces the enzymatic activity of LDH.

### *pdhC* is a potential target mRNA of SmsR1, affecting global protein acetylation level

**Fig 6:**
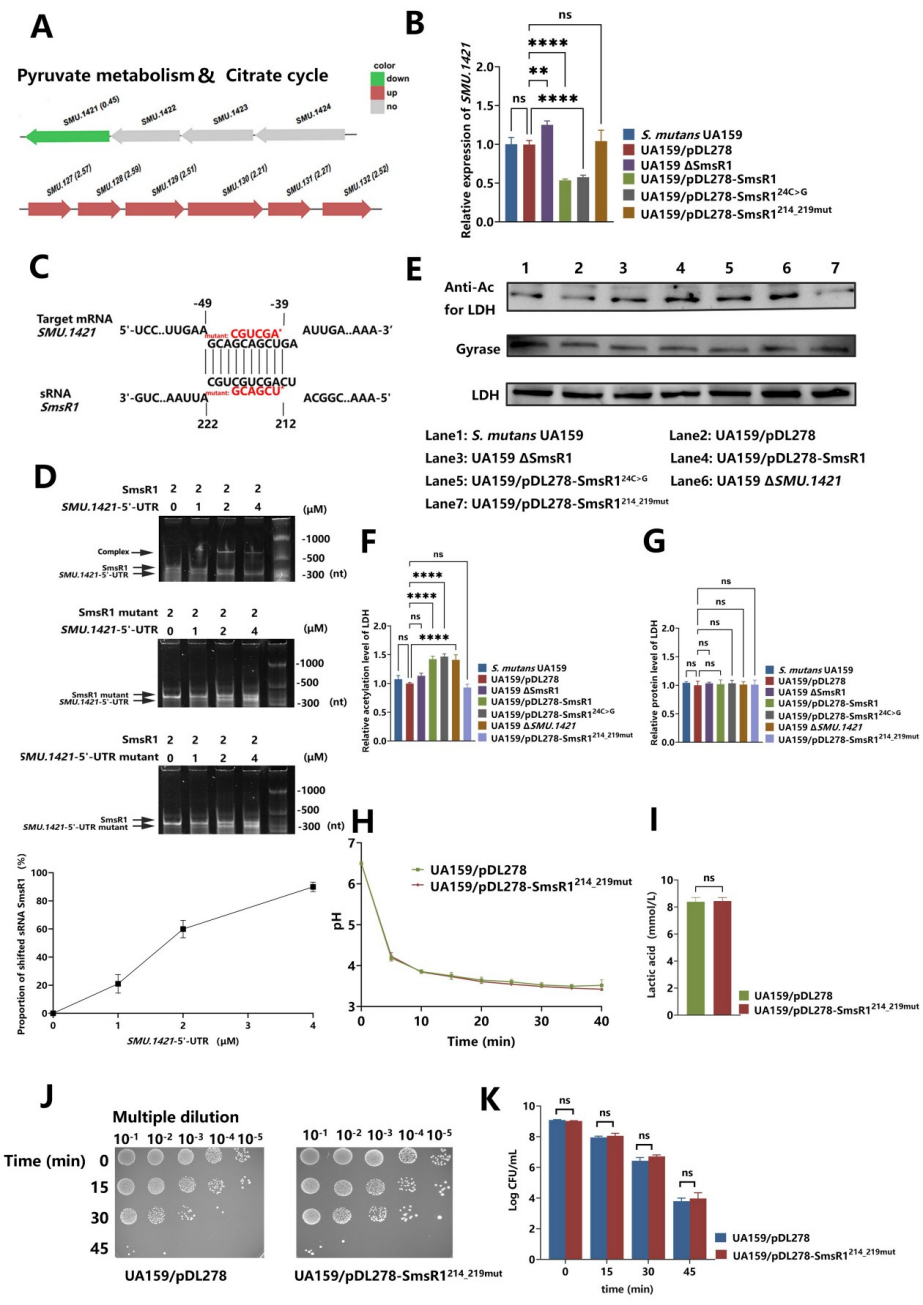
Effect and interaction of SmsR1 on the potential target mRNA *pdhC* and acetylation level. (A) Transcriptome results of the SmsR1 overexpressing strain. (B) *pdhC* mRNA level was determined by qRT-PCR using the 2 -^ΔΔCT^ method. (C) Predicted base pairing sequence between sRNA SmsR1 and target mRNA *SMU*.*1421*(*pdhC*), and the motifs of point mutations are shown in red. (D) The interaction between sRNA SmsR1 and its mutant with the target mRNA *SMU*.*1421*- 5’ UTR and its mutant was determined by R-EMSA experiments *in vitro*, and the proportion of shifted sRNA SmsR1changed with the increase of *SMU*.*1421*- 5’ UTR concentration. (E) The acetylation level of LDH was detected by anti-acetyl lysine western blotting. (F) Anti-acetyl lysine of LDH bands were quantified with ImageJ software and normalized to the control group. (G) Anti-LDH bands were quantified with ImageJ software and were normalized to the control group. (H) pH values of culture supernatants of UA159/pDL278 and UA159/pDL278-SmsR1^214_219mut^ treated with 1% glucose for 40 minutes. (I) The lactic acid production of UA159/pDL278 and UA159/pDL278-SmsR1^214_219mut^. (J) Survival of UA159/pDL278 and UA159/pDL278-SmsR1^214_219mut^ after acid killing with glycine at pH = 2.8 for 0, 15, 30 and 45 minutes. (K) CFU numbers of surviving cells in strains after acid killing treatment. Results are expressed as mean and SD; n = 3 biological replicates, statistical significance was determined using one-way analysis of variance (ANOVA) and Tukey’s test were performed to compare data between multiple groups; *P* < 0.05 was considered statistically significant. (ns, *P* > 0.05; *, *P* <0.05; **, *P* < 0.01; ***, *P* <0.001; ****, *P* <0.0001).

Strains overexpressing SmsR1 and SmsR1^24C>G^ showed increased Ac-CoA and acetylation levels that led us to suspect that SmsR1 caused this series of changes through a target gene. To explore how SmsR1 is involved in the post-translational modification of acetylation, we again reviewed the overlapping results of the transcriptome and the target genes predicted by *IntaRNA*. We identified that the target gene *SMU*.*1421* (*pdhC*) which can transfer acetyl to CoA to form Ac-CoA was significantly down-regulated (Fig 6A and 6B). According to previous studies, *pdhC* is regulated by the pdh operon (*pdhD-pdhA-pdhB-pdhC*), PdhC is the E2 component of the four-enzyme pyruvate dehydrogenase complex which catalyzed the pyruvate decarboxylation reaction while transferring the acetyl to CoA to generate Ac-CoA [40]. However, of these only *SMU*.*1421* (*pdhC*) had significant changes (Fig 6A). This suggested that the downregulation of *SMU*.*1421* (*pdhC*) in strains overexpressing SmsR1 and SmsR1^24C>G^ was not caused by pdh operon, but probably by the direct base pairing with sRNA SmsR1 (Fig 6C). In addition, ADH and neighboring genes (*SMU*.*127* to *SMU*.*132*) of the same family as PDH were significantly upregulated upon SmsR1 overexpression (Figs 6A and S4A), potentially leading to increased acetylation levels compensatively.

To verify this conjecture, we transcribed and synthesized the sRNA SmsR1, mutated SmsR1 (the motif AGCUGC was mutated to UCGACG), the 5’-UTR of *SMU*.*1421* mRNA and mutated 5’-UTR of *SMU*.*1421* (the motif GCAGCU was mutated to CGUCGA) in vitro, and successful transcription was verified on a 2% TBE agarose gel. On the premise that each RNA alone does not drive the formation of large molecular self-interactions (S5 Fig), RNA-RNA EMSA experiments were conducted *in vitro*, and the results showed that sRNA SmsR1 and 5’-UTR of *SMU*.*1421* could indeed bind together, and after point mutation, this binding could not be observed (Fig 6D). Next, we knocked out the *pdhC* gene (UA159 Δ*SMU*.*1421*), and found that the mRNA levels of *SMU*.*127* to *SMU*.*132*. were higher upon *SMU*.*1421* deletion (S4B Fig). The LDH acetylation levels of SmsR1 overexpression and *SMU*.*1421* deletion strains were relatively increased (Fig 6E, 6F, and 6G).

Meanwhile, we constructed a mutant variant of pDL278-SmsR1 named pDL278-SmsR1^214_219mut^ (the motif AGCTGC was mutated to TCGACG) and introduced it into UA159, and its acetylated level of LDH returned to the same as UA159/pDL278 (Fig 6E, 6F, and 6G). We repeated the acidogenicity (Fig 6H and 6I) and ATR (Fig 6J and 6K) phenotype tests. The phenotypes of UA159/pDL278-SmsR1^214_219mut^ in acidogenicity or acid tolerance showed no significant difference compared to the control group.

### SmsR1 overexpression weakened *S*. *mutans* cariogenic virulence

**Fig 7:**
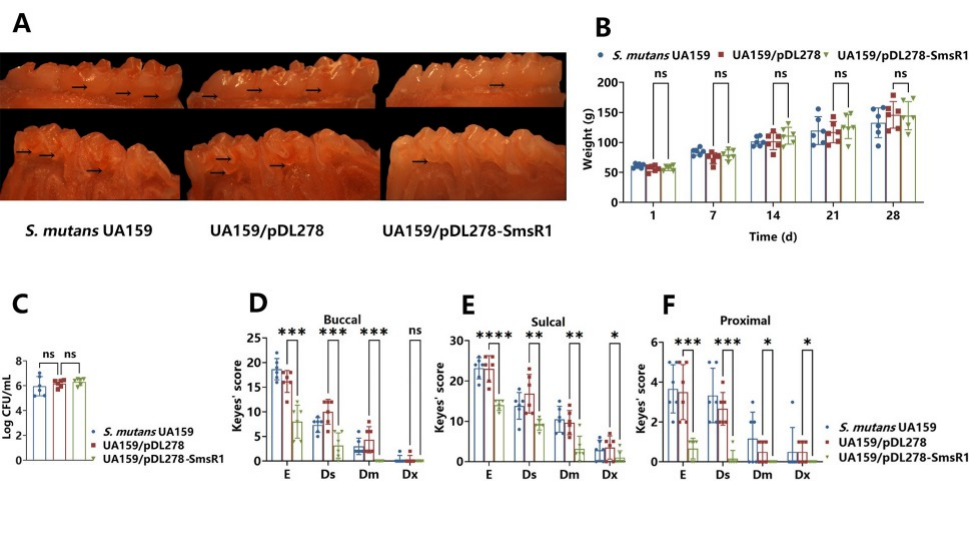
SmsR1 overexpression affects cariogenic virulence in vivo. (A) Representative images showing the buccal (upper) and sulcal (lower) sides of the molars. Black arrows indicate representative caries lesions. (B) The body weights of rats as recorded until the end of the experiment. (C) At the end of the experiment, the dental plaque of each group of rats was taken and the CFU was counted. Keyes’ scores are shown for the buccal (D), sulcal (E), and proximal (F) surfaces of each group (n = 6). The severity of caries lesions as evaluated for the following four types: enamel only (E), slight dentin (Ds), moderate dentin (Dm), and extensive dentin (Dx). Statistical significance was determined using one-way analysis of variance (ANOVA) and Tukey’s test were performed to compare data between multiple groups; *P* < 0.05 was considered statistically significant. (ns, *P* > 0.05; *, *P* <0.05; **, *P* < 0.01; ***, *P* <0.001; ****, *P* <0.0001).

*S*. *mutans* reduces the pH of the oral environment by fermenting dietary carbohydrates to produce acidic substances such as lactic acid and formic acid, which demineralize the tooth surface. Therefore, acidogenicity is the direct cause of cariogenic virulence. To further determine the cariogenic effect of sRNA SmsR1 on *S*. *mutans*, we constructed rat caries animal models infected with control and SmsR1 overexpressing strains (Fig 7A). To detect the effect of *S*. *mutans* on the health status of rats, we recorded the body weight of rats every alternate day. The results showed no significant difference among all groups (Fig 7B). We collected dental plaque from the rats in the beginning, middle, and end of the experiment and performed PCR detection to ensure that the bacterial inoculation was successful and the overexpressed plasmid was present in *S*. *mutans*. After the experiment, the dental plaque of rats was screened with SB-10 agar and the CFUs were determined (Fig 7C). The results showed no statistical difference in the number of *S*. *mutans* among all groups (*P* > 0.05), indicating that the difference in cariogenic virulence was not due to the extent of *S*. *mutans* colonization.

We used the Keyes’ score to assess the depth and severity of carious lesions. The evaluation items included enamel only (E), slight dentinal (Ds), moderate dentinal (Dm), and extensive dentinal (Dx) [41]. No significant difference in caries depth and severity was observed between the two control groups from the buccal, sulcal and proximal surfaces as shown in Fig 7D, 7E, and 7F (*P* > 0.05). However, the degree of carious lesions of the SmsR1 overexpressing group in E, Ds and Dm was significantly lower than that of the empty vector control as assessed from the buccal surface (Fig 7D). Similarly, the results of sulcal and proximal surface observations also showed that the degree of carious lesions of the SmsR1 overexpression group in E, Ds, Dm and Dx was significantly lower than the empty vector control (Fig 7E and 7F). From the results of caries animal experiments in rats, we conclude that SmsR1 overexpression weakens the cariogenic virulence of *S*. *mutans*.

## Discussion

The survival and adaptability of bacteria in dynamic environments largely depends on intricate regulatory networks, ranging from genetic to post-translational levels, that coordinate various cellular processes. In this study, we investigated the role of the sRNA SmsR1 in the cariogenic bacterium, *S*. *mutans*, a major contributor to dental caries. Previous studies predominantly focused on sRNA-mediated post-transcriptional regulation, where sRNAs often bind target mRNAs to influence their stability or translation. Our study lends support to this notion, revealing that SmsR1 not only partakes in these classical sRNA functions but also bridges post-transcriptional and post-translational regulations, specifically lysine acetylation in *S*. *mutans*.

**Fig 8:**
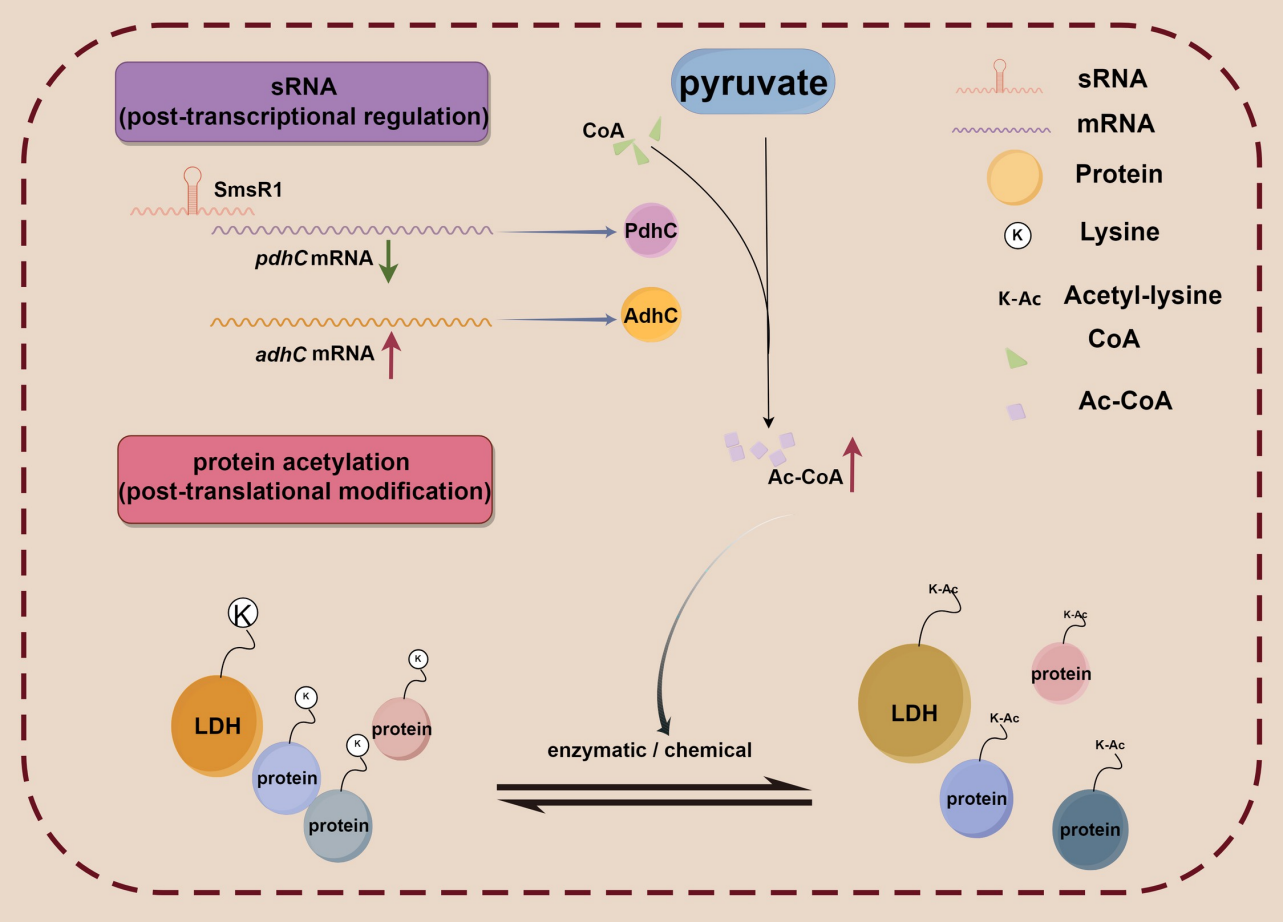
Schematic representation of the possible mechanism of post-transcriptional regulation of SmsR1 participating in the acetylation of post-translational modifications in *S*. *mutans* UA159. Overexpression of SmsR1 interferes with target mRNA *pdhC* expression through base pairing, resulting in the disruption of the homeostasis of Ac-CoA. Ac-CoA, the main donor of acetylation increases, leading to changes in acetylation and redistribution of acetylation levels. The acetylation levels of LDH and other proteins increase, and the enzymatic activity of LDH decreases after acetylation. This Figure was created using Figdraw (www.figdraw.com).

Using Krieger MC’s research as a foundation for our study [30], we generated 10 sRNA overexpression strains. The results from the analysis of these strains highlighted the influence of SmsR1 on the acidogenicity of *S*. *mutans*, a critical determinant of its cariogenic potential. Surprisingly, SmsR1 does not merely exert its effect by modulating mRNA stability. Instead, it appears to integrate regulatory signals at the post-transcriptional and post-translational levels, allowing the bacterium to swiftly and efficiently respond to environmental stresses (Fig 8).

The decreased acidogenicity that we observed upon SmsR1 overexpression stems primarily from reduced lactate production due to diminished LDH activity. This decline in LDH activity is intriguingly not attributed to reduced *ldh* mRNA levels but rather to increased acetylation of the enzyme, a post-translational modification known to inhibit its function. Such a modification depends on the availability of the acetyl donor, Ac-CoA. There was a significant increase in Ac-CoA levels in the SmsR1 overexpressing strain, which we traced back to SmsR1-mediated repression of *pdhC* (*SMU*.*1421*) mRNA, a critical component of the pyruvate dehydrogenase complex, responsible for Ac-CoA production. Previous studies have shown that *pdh* is an operon consisting of *pdhD-pdhA-pdhB-pdhC* and is important for the survival of sugar-starved *S*. *mutans* [40]. This repression not only signifies SmsR1’s regulatory footprint on metabolic pathways but also its indirect influence on protein acetylation patterns, emphasizing the multifaceted nature of sRNA-mediated regulation.

In our results, we observed down-regulation of only *pdhC* and up-regulation of Ac-CoA. This was contrary to our expectation given that *pdhC* is essential in Ac-CoA production through its role as PDH. However, further investigation revealed a feedback mechanism where reduced *pdhC* levels enhance other metabolic pathways, culminating in elevated Ac-CoA levels. We observed that acetoin dehydrogenase (ADH), a member of the 2-oxoacid dehydrogenase family with physiological functions similar to PDH, exhibited a significant increase in gene expression upon SmsR1 overexpression. This upregulated expression of *adhC* was more obvious in UA159 Δ*SMU*.*1421*, which contributing to the transfer of acetyl groups to CoA to form Ac-CoA. Our results suggest a potential cascade effect: Upon artificial overexpression of SmsR1 (S6 Fig), we found *pdhC* expression to be significantly inhibited. This causes a disruption in the balance between *pdhC* and *adhC* expression levels and creates a compensatory effect. Specifically, the compensatory increase in *adhC* results in elevated Ac-CoA production, contributing to an increase in available acetylation donors within the bacterial cell. This shift in the overall acetylation level distribution likely affects various target proteins, including lactate dehydrogenase (LDH), leading to an increase in LDH acetylation and a subsequent decrease in its enzymatic activity.

The exceptional ability of *S*. *mutans* to establish and maintain its ecological dominance in the ever-fluctuating pH environment of the oral cavity is primarily attributed to its acid tolerance response (ATR) [42]. *S*. *mutans* attempts to maintain an intracellular pH higher than the extracellular environment (termed ΔpH), thereby preventing damage to acid-sensitive DNA and enzymes [43,44]. The molecular machinery regulating ATR in *S*. *mutans* is intricate, involving a diverse array of genes and associated pathways spanning an estimated 10–20% of the genome being involved [45,46]. Consistent with previous studies, our results reveal the roles of pdhC and LDH in potentially attenuating the acid tolerance capacity upon SmsR1 overexpression. Deletion of *pdhA*, the E1α subunit of PDH, increased the sensitivity of *S*. *mutans* to acid stress [47]. LDH is a key enzyme in *S*. *mutans* lactic acid efflux, proton removal, and maintenance of pH homeostasis [48]. Our data provide evidence for increased LDH acetylation, accompanied by a concurrent decline in its enzymatic activity, potentially contributing to the observed decreased acid tolerance phenotype. The coordination of acid production and tolerance in *S*. *mutans* provides the bacterium with a dynamic toolkit to navigate the challenging pH landscape of the oral environment.

sRNAs usually lack the ability to encode proteins [49]. Interestingly, we identified the coding sequence for ComS, a 17-amino acid peptide of the quorum system ComRS within SmsR1 and the precursor of the signal peptide XIP. The ComRS quorum sensing systems appear to be activated by different environmental conditions, nutrient-rich media like BHI may inhibit the activity of ComRS. In contrast, the sensitivity of the ComRS system is significantly more profound in chemically defined media (CDM) compared to rich media [31,32,50]. This poses potential challenges in delineating the regulatory role of SmsR1 purely as an RNA molecule. Therefore, to eliminate the potential interferences from the protein-coding ComS region within SmsR1, we strategically employed BHI rich broth, which was also the same as the condition where SmsR1 was initially identified [30], and it seems that ComS did not play a dominant role under this condition. Results show that SmsR1 overexpression in BHI broth results in weaker acid production via base pairing, indicating no obvious relationship with ComS validating our experimental approach. We constructed overexpression mutants and deletion mutants, with and without ComS, and even point mutation mutants by mutating 24 site C into G to form the stop codon of TGA in ComS to nullify its protein-coding capacity. These mutations reveal the function of only SmsR1-mediated target mRNA regulation by base pairing in regulating acidogenicity of *S*. *mutans*. There is no significant overlap of SmsR1 with exogenously added XIP to *S*. *mutans* in transcriptome results [51]. These results indicate that SmsR1 may serve as a dual-functional sRNA performing a regulatory role through base pairing and as a potential protein-coding entity for ComRS in *S*. *mutans*, similar to the dual-function sRNAs recently reported in other bacteria [52,53]. For instance, RNAIII regulates some mRNAs encoding virulence factors via direct base pairing in *Staphylococcus aureus* [54,55], and also encodes δ-hemolysin (a 26-amino acid secreted peptide) independent of the target mRNAs [56]. In recent research, novel dual-function RNAs SgrS, Spot 42 and AzuCR have also been described [36,37,57–59]. These evidences suggest that SmsR1 is possibly also a dual-functional RNA, which can not only perform through base pairing but also encode the peptide ComS to regulate the physiological functions of *S*. *mutans*. However, whether the two functions of SmsR1 coordinate or interfere with each other and the timing of trigger of their respective functions are still open questions that require further research.

In this study, we found that SmsR1 can significantly weaken the acidogenicity in *S*. *mutans* and that the cariogenic virulence was also greatly reduced in animal models. sRNA has attracted attention as a novel strategy for RNA drugs to fight pathogens [60], and regulating bacterial virulence through modulation of acetylation levels presents a promising therapeutic strategy. sRNA often regulates multiple target genes, and SmsR1 may have other potential targets awaiting our identification [61,62]. In conclusion, our study highlights the intricate web of regulatory mechanisms employed by *S*. *mutans*, with SmsR1 emerging as a link between post-transcriptional and post-translational regulation. By unveiling the cross-talk between sRNA-mediated regulation and lysine acetylation, we gain deeper insights into the molecular underpinnings of bacterial adaptability and pathogenicity. Such revelations not only enrich our basic understanding of bacterial physiology but also point towards novel therapeutic strategies to tackle bacterial diseases.

## Material and methods

### Ethics statement

The animal experiments in this study were performed in strict accordance with the protocols and procedures approved by the Institutional Animal Care and Use Committee of West China Hospital of Stomatology, Sichuan University (WCHSIRB-D-2021-155). The animal care and protocols adhered to the Chinese National Laboratory Animal Guidelines for Ethical Review of Animal Welfare. Our experiments meet the humane standards and minimizes the animals’ pain.

### Bacterial strains, plasmids, and primers

The bacterial strains, plasmids, and primers used in this study are listed in S5 Table. The *S*. *mutans* UA159 strain mutants were constructed as follows, the deletion mutant strains (UA159 Δ*SMU*.*1421*) were generated using a two-step transformation procedure with markerless in-frame deletion method [63] and then selected on BHI agar plates containing 4 mg/mL *p*-CL-Phe (Sigma, USA). The overexpression strains UA159/pDL278-SmsR1, UA159/pDL278-SmsR1P1, and UA159/pDL278-SmsR1P2 were constructed by introducing the *ldh* promoter and gene onto the *E*. *coli-Streptococcus* shuttle vector pDL278. The PCR products and vectors were digested using restriction enzymes EcoRI and BamHI (Takara, Japan), and then ligated using T4 ligase to generate the plasmid clones. The point mutations vector pDL278*-*SmsR1^24C>G^ and pDL278-SmsR1^214_219mut^ were generated using the In-Fusion HD cloning kit (TaKaRa, Japan) from the pDL278*-*SmsR1 plasmid. The plasmid clones were transformed into the *S*. *mutans* UA159 strain and selected on BHI agar plates containing 1.0 mg/mL spectinomycin. All PCR products and mutants were verified by PCR and DNA sequencing.

### Bacterial growth and culture conditions

*S*. *mutans* UA159 and its derivatives were grown in brain heart infusion (BHI) broth (BD, USA) or on BHI agar at 37°C in anaerobic conditions (85% N_2_, 10% H_2_, 5% CO_2_). BHI broth supplemented with 1% sucrose (1% BHI-s) was used to cultivate *S*. *mutans* biofilms. *Escherichia coli* DH5α was inoculated in Luria-Bertani medium (1% NaCl, 1% tryptone, 0.5% yeast extract) under aerobic conditions. When required, spectinomycin was added to growth media at the following concentrations: 1.0 mg/mL for *S*. *mutans* and 100 μg/mL for *E*. *coli*.

For plaque samples collected from rats, SB-10 agar plates containing bacitracin (0.2 U/mL) were used for *S*. *mutans* screening and colony-forming unit (CFU) determination as described earlier with slight modifications [64].

### Growth curves

Overnight suspension cultures were diluted 1:10 into fresh BHI broth and then grown to OD _600_ = 0.5 to ensure that the bacteria were in the exponential phase. Bacteria were then diluted to 1:100 in fresh BHI (pH = 7 or 5.5) broth. The diluted cultures were transferred into 96-well flat-bottom microplates and overlaid with mineral oil to seal the liquid surface. A microplate reader (BioTek, USA) detected OD _600_ and plotted the growth curve at an interval of 30 minutes. Each experiment was performed in three replicates.

### Biofilm formation assays

*S*. *mutans* strains were cultured overnight, diluted 1:10 in fresh BHI broth, grown to OD _600_ = 0.5 and then diluted 1:100 in fresh 1%BHI-s (pH = 7 or 5.5) broth. The culture medium was divided into 96-well microplates and cultured for 24h under anaerobic conditions for biofilm formation. The biofilm was quantified by crystal violet (CV) assays. Briefly, the medium was discarded after culture and rinsed twice with PBS to remove planktonic bacteria. The wells were fixed with methanol for 15 minutes and then air-dried naturally. After staining with 0.01% CV for 15 minutes, the dyes were washed off with PBS and the pellets were redissolved in 33% glacial acetic acid. The OD _600_ of the dissolved solution was recorded. Water-insoluble exopolysaccharides (EPS) were measured by sulfuric anthrone reaction [65], and the value of OD _625_ was recorded.

### Glycolytic pH drop and biofilm lactic acid production assays

Evaluation of the effect of SmsR1 on glycolysis of *S*. *mutans* was performed by pH drop experiment as per Xu’s method with slight modification [66]. As mentioned above, overnight cultures were subcultured in BHI broth to exponential phase (OD_600_ = 0.5). The cultures were centrifuged (4000 g, 10 minutes) to harvest the cell pellets and rinsed with potassium phosphate buffer (pH = 6.5, 37.5mM KCl and 1.25mM MgCl_2_). The cell pellets were resuspended in PPB containing 1% glucose (W/V), and the pH values were recorded by a Benchtop meter (Thermo Scientific, Waltham, MA) every 5 minutes for 40 minutes.

The acidogenicity of *S*. *mutans* biofilm was evaluated using a lactic acid kit (Jiancheng, Nanjing, China). *S*. *mutans* was diluted using 1% BHI-s broth to 1:100 and cultured in 24-well plates under anaerobic conditions for 24 hours to form biofilms. The culture medium was discarded and rinsed thrice with PBS. The cells were then incubated with buffered peptone water (BPW, HuanKai, Guangzhou, China) containing 0.2% sucrose (W/V) at 37°C for 2 hours anaerobically to produce lactic acid. Following the kit instructions, OD _570_ was measured after the reagent reacted with the supernatant. The amount of lactic acid produced was calculated using standard curves.

### Lactate dehydrogenase enzymatic activity assays

20 mL OD_600_ = 0.5 of *S*. *mutans* was harvested and total protein was extracted by mechanical cell wall lysis at 4°C. Total protein concentration was estimated by the BCA protein assay kit using a part of the liquid (Beyotime, Shanghai, China) while the remaining was used to measure LDH activity by an LDH enzymatic activity kit (Solarbio, Beijing, China) as per the manufacturer’s instructions. The differences in LDH enzymatic activities were compared based on the control group.

### Acid killing assays

The culture conditions and harvesting methods of *S*. *mutans* were the same as described above. The acid tolerance of *S*. *mutans* was evaluated by treating the cells with glycine (pH = 2.8) as described by Zhang et al [67]. After treatment for 15, 30 and 45 minutes, the acid-killing treatment was terminated with PBS and the reaction solutions were serially diluted. 5 uL of the dilutions were spotted on BHI agar plates and cultured anaerobically at 37°C for 72 h. The CFU of each group was counted to estimate acid killing.

### RNA-seq and other bioinformatic tools

50 mL cultures of UA159/pDL278 and UA159/pDL278*-*SmsR1 at OD _600_ = 0.5 were grown in BHI broth for transcriptome analysis and processed as previously described [68]. Bacterial cells were harvested by centrifugation and snap-frozen in liquid nitrogen for 10 minutes and promptly stored at -80°C. Three replicates of each group were subsequently submitted to Majorbio Co., Ltd. (Majorbio, China) for whole-genome RNA sequencing. Total RNA was extracted using TRIzol Reagent following the manufacturer’s protocol (Invitrogen). Genomic DNA was removed using DNase I (Takara, Japan). Genes with expression fold change of 2.0 and *P* value < 0.05 were considered as differentially expressed genes (DEGs). Data analysis was performed using the online platform of Shanghai Majorbio Biomedical Technology Co., Ltd.’s Majorbio cloud platform (www.majorbio.com). The DEGs were further used for KEGG pathway analysis (https://david.ncifcrf.gov/).

The intrinsic terminator of SmsR1 was predicted by the ARNold Web Server (http://rssf.i2bc.paris-saclay.fr/toolbox/arnold/). *IntaRNA* (IntaRNA—RNA-RNA interaction, uni-freiburg.de) was used to predict the top 100 target mRNAs of SmsR1 in *S*. *mutans* UA159 by searching +75 nt ~ -75 nt. The results are shown in S2 Table. Potential target mRNAs were screened based on transcriptome results, bioinformatics analysis, and prediction results.

### Acetyl-coenzyme A (Ac-CoA) ELISA assay

10 mL of *S*. *mutans* culture at OD _600_ = 0.5 was collected and total protein was extracted by mechanical cell wall lysis at 4°C. The protein concentration was measured using a BCA protein assay kit (Beyotime, Shanghai, China) as described above. A double antibody sandwich enzyme-linked immunosorbent assay (ELISA) kit (JININGSHIYE, Shanghai, China) was used to determine the level of Ac-CoA in protein samples as per the manufacturer’s instructions. The color intensity of the reaction solution was positively correlated with the Ac-CoA in the sample. OD _450_ values were measured using a microplate reader and the Ac-CoA contents were calculated using standard curves.

### Acetyl-proteomics

Cells from 300 mL cultures of UA159/pDL278 and UA159/pDL278-SmsR1 at OD _600_ = 0.5, cultivated at pH 7 were collected by centrifugation, snap-frozen in liquid nitrogen for 10 minutes, and promptly submitted to PTM BIO Co., Ltd. (Hangzhou, China) for acetyl-proteomics analysis. To enrich modified peptides, tryptic peptides dissolved in NETN buffer (100 mM NaCl, 1 mM EDTA, 50 mM Tris-HCl, 0.5% NP-40, pH 8.0) were incubated with pre-washed antibody beads (PTM Bio) at 4°C overnight with gentle shaking. The beads were then washed four times with NETN buffer and twice with H_2_O. The bound peptides were eluted from the beads with 0.1% trifluoroacetic acid. Finally, the eluted fractions were pooled together and vacuum dried. For LC-MS/MS analysis, the resulting peptides were desalted with C18 ZipTips (Millipore) according to the manufacturer’s instructions. Results from the LC-MS/MS analysis were analyzed by database searching and annotation, and proteins with expression fold changes of 1.1 were considered as differentially modified proteins. Gene Ontology and KEGG pathway analysis were used for further hierarchical clustering based on differentially expressed protein functional classification.

### RNA extraction and qRT-PCR

Total RNA was extracted from *S*. *mutans* using MasterPure Complete DNA and RNA Purification Kit (Epicentre, USA). Genomic DNA was then eliminated and double-stranded cDNA synthesized using PrimeScript RT reagent Kit with gDNA Eraser (Takara, Japan). Quantitative real-time PCR (qRT-PCR) was then performed using TB Green Premix Ex Taq II mix (Takara, Japan) on an Applied Biosystems QuantStudio 6 unit (Thermofisher, USA). Validated primers used in qRT-PCR are listed in S5 Table. Data were collected using QuantStudio Real-Time PCR Software v1.3, normalized to reference gene 16S rRNA levels, and analyzed using the 2-^ΔΔCT^ method. Each experiment was performed in triplicate.

### Western blotting

Total protein extract for Western blotting was prepared as described previously [69], and the protein concentrations were determined using a BCA protein assay kit. In each experiment, we ran identical SDS-PAGE gels simultaneously (proteins from the same batch of samples, same loading amount), one gel was used for Coomassie staining, to ensure equal total protein amounts, and the others were used for Western blotting. Equal amounts of protein (50 μg) were incubated with SDS-PAGE Protein Loading Buffer (YEASEN, China) at 99°C for 10 minutes. The samples were resolved on a 4–20% SDS-PAGE gradient Gel (YEASEN, China) at 110 V and transferred to a polyvinylidene difluoride (PVDF) on the ice. Membranes were blocked with 5% skim milk in TBST at room temperature for 1 hour. Subsequently, the membranes were incubated with anti-acetyl lysine antibody (Anti-Ac,1:1000), anti-Gyrase (1:1000) and anti-LDH (1:1000) in TBST containing 5% skim milk at 4°C overnight. Membranes were then washed in TBST and incubated in HRP conjugated goat anti-mouse (for Anti-Ac) and goat anti-rabbit (for anti-Gyrase and anti-LDH) secondary antibody diluted to 1:10000 in the blocking solution above for 2 hours. Membranes were visualized with an immobilon Western Chemiluminescent HRP substrate kit (Millipore) on ChemiDoc Imaging Systems (Bio-Rad, USA), and ImageJ software was used to analyze band intensities. The prominent band was subsequently submitted to PTM BIO Co., Ltd. (Hangzhou, China) for identification by liquid chromatography-tandem mass spectrometry (LC-MS/MS).

### *In vitro* transcription and RNA-RNA electrophoretic mobility shift assay

The *in vitro* transcription DNA templates of SmsR1, and *SMU*.*1421* 5’ UTR mRNA (300nt length including the binding region) were PCR amplified using the *S*. *mutans* UA159 genome as the template. The DNA templates of SmsR1 mutant and *SMU*.*1421* 5’ UTR mutant were carried on pUC57 plasmid and constructed by Tsingke (Tsingke, China). The DNA templates were then transcribed into SmsR1, mutant SmsR1, and *SMU*.*1421* 5’ UTR, mutant *SMU*.*1421* 5’ UTR using the Hifair T7 High Yield RNA Synthesis Kit (YEASEN, China). The RNAs were then purified using the Hieff NGS RNA Cleaner (YEASEN, China). An RNA-RNA electrophoretic mobility shift assay (R-EMSA) was performed to detect the sRNA-mRNA interactions, as previously described with some modifications [70, 71]. R-EMSAs were performed with SmsR1, SmsR1 mutant (2 μM), and *SMU*.*1421* 5’ UTR, *SMU*.*1421* 5’ UTR mutant mRNA (0,1, 2, and 4 μM) in R-EMSA binding buffer (20 mM Tris–HCl, pH 8.0, 1 mM DTT, 1 mM MgCl_2_, 20 mM KCl, 10 mM Na_2_HPO4-NaH_2_PO4, pH 8.0). The reaction mixtures were incubated at 37°C for 15 minutes. A 4% polyacrylamide native gel for R-EMSA was prepared by mixing 1mL 30% acrylamide solution, 1.5 mL 2.5× TBE, 1.25 mL 50% glycerol, and 4.25 mL RNase-free water. To this 37.5 μL of 10% APS and 8 μL of TEMED was added to form a 100 mm × 75 mm × 1 mm gel. 0.5× TBE containing 5% glycerol was used as a running buffer, and the gel was run at 20 mA until the bromophenol blue migrated to 2/3 of the gel. The gels were stained with GelRed nucleic acid gel stain (Tsingke, China) for 10 minutes, and immediately visualized with UV light on ChemiDoc Imaging Systems (Bio-Rad, USA). All buffers and solutions were prepared with RNase-free water.

### Rat caries model of *S*. *mutans* infection

The rat caries model was established as described with some modifications [72,73]. 18 female specific-pathogen-free (SPF) Sprague-Dawley rats aged 3 weeks were purchased from Dossy Inc (Chengdu, China). The rats were given ddH_2_O containing ampicillin (1g/L) for the first three days, and then ddH_2_O for another day to wash away the ampicillin. Next, the rats were randomly divided into three groups (n = 6), and infected by oral swabbing with BHI broth containing UA159, UA159/pDL278, or UA159/pDL278-SmsR1 (OD _600_ = 0.5) for three days. The infection was confirmed by culturing dental plaque on the SB-10 agar plates and by PCR validation. Simultaneously, the rats were fed the cariogenic diet 2000 (Trophic Diet, Nantong, China) and drank dd H_2_O containing 5% sucrose (W/V). The rats were weighed every alternate day throughout the experiment. After feeding for three weeks, the rats were euthanized by carbon dioxide asphyxiation. Their maxillae and mandibles were collected and dental plaque samples were sonicated in 5 mL sterile PBS. The obtained suspensions were plated on SB-10 agar with bacitracin for CFU measurement to estimate the *S*. *mutans* colonization. Presence of UA159/pDL278 and UA159/pDL278-SmsR1 plasmids was verified by PCR. Molar teeth of rats were stained with 0.4% murexide overnight away from light in a dark place. Images were taken under a stereomicroscope (Leica, Heerbrugg, Switzerland) and dental caries and severity were evaluated by the Keyes’ method [41].

### Statistical analysis

All assays were performed in triplicate and reproduced at least three times. The obtained data were statistically analyzed using SPSS 25.0 (SPSS Software Inc, Chicago, IL, USA) and plotted with GraphPad Prism 8.0.2 software (GraphPad Software Inc, San Diego, CA, USA). Results were presented as means and standard deviations. The unpaired Student’s t-test was applied to compare data between two groups, determined using one-way analysis of variance (ANOVA), and Tukey’s test was performed to compare data between multiple groups. A *P* value of 0.05 was considered statistically significant. Significance was indicated in the figures by asterisks (*, *P* <0.05; **, *P* < 0.01; ***, *P* <0.001; ****, *P* <0.0001).

## Supporting information

S1 Table Transcriptome results of UA159/pDL278 and UA159/pDL278-SmsR1. (XLSX)

S2 Table Predicted target mRNAs of sRNA SmsR1 by *IntaRNA*. (XLSX)

S3 Table The up-regulated acetyl-proteomics result of UA159/pDL278 and UA159/pDL278-SmsR1. (XLSX)

S4 Table The LC-MS/MS identification results of the prominent band extracted from SDS-PAGE. (XLSX)

S5 Table Bacterial strains, plasmids and primers used in this study. (XLSX)

S1 Fig pH values of culture supernatants of the 10 overexpression strains treated with 1% glucose for 40 minutes. (TIF)

S2 Fig The total protein electrophoresis patterns of SDS-PAGE were consistent among the four strains. (TIF)

S3 Fig The prominent band was identified by LC-MS/MS, and a characteristic peptide map of LDH is displayed. (TIFF)

S4 Fig The mRNA levels of *SMU*.*127-SMU*.*132* in UA159/pDL278, UA159/pDL278-SmsR1 (A) and UA159 Δ*SMU*.*1421* (B) as evaluated by qRT-PCR using the 2 ^- ΔΔCT^ method.
(TIF)

S5 Fig Bands of each RNA alone at each of the concentrations. (TIF)

S6 Fig The mRNA levels of SmsR1 are increased nearly 100-fold in UA159/pDL278-SmsR1. (TIF)
