# Supplementary figures and images for: Small RNA SmsR1 modulates acidogenicity and cariogenic virulence by affecting protein acetylation in Streptococcus mutans
Source: PLoS Pathog. 2024 Apr 15;20(4):e1012147. doi: 10.1371/journal.ppat.1012147 (PMC11045139; doi:10.1371/journal.ppat.1012147)

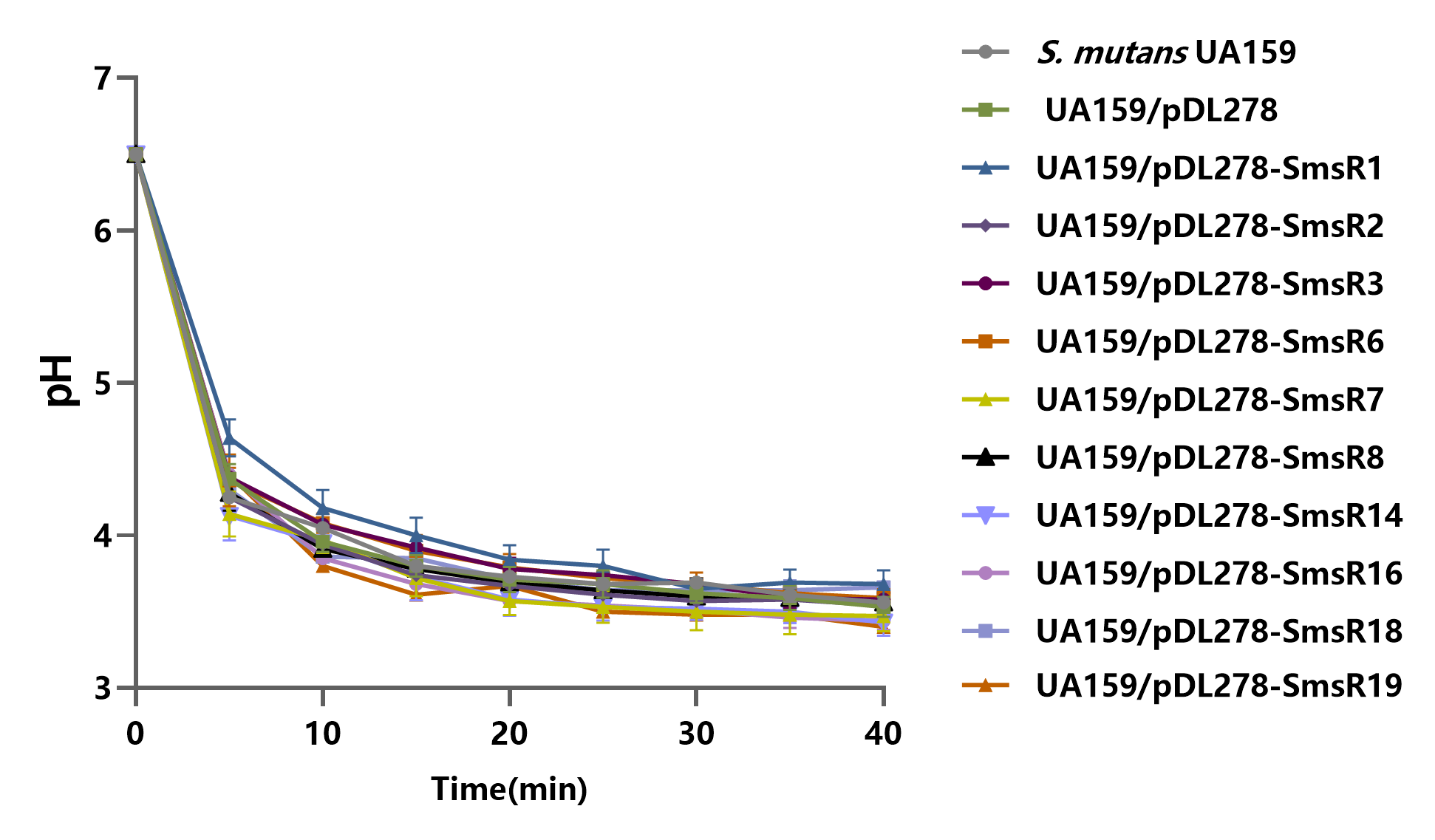

Supplement: S1 Fig — (TIF) [file ppat.1012147.s006.tif]

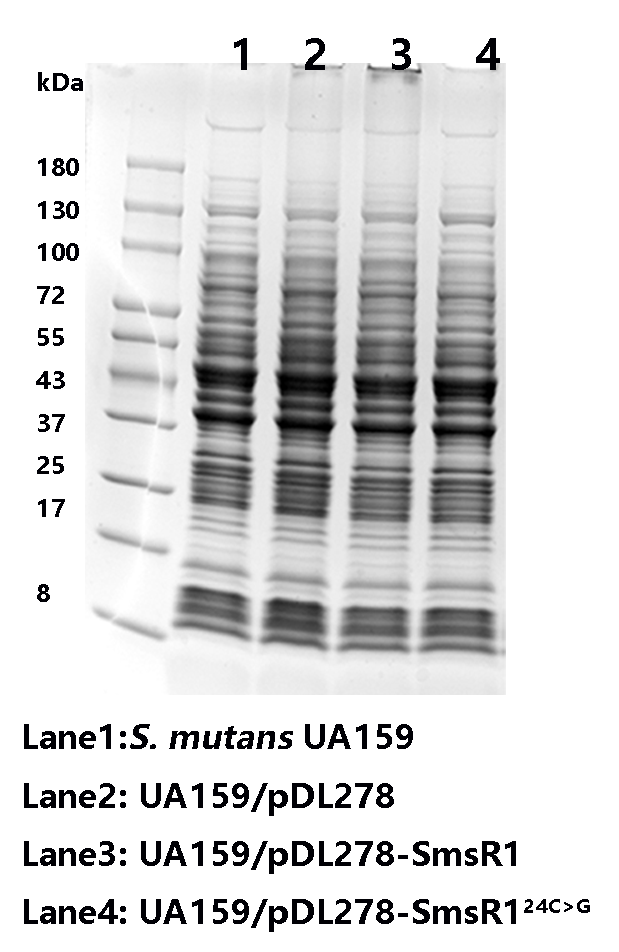

Supplement: S2 Fig — (TIF) [file ppat.1012147.s007.tif]

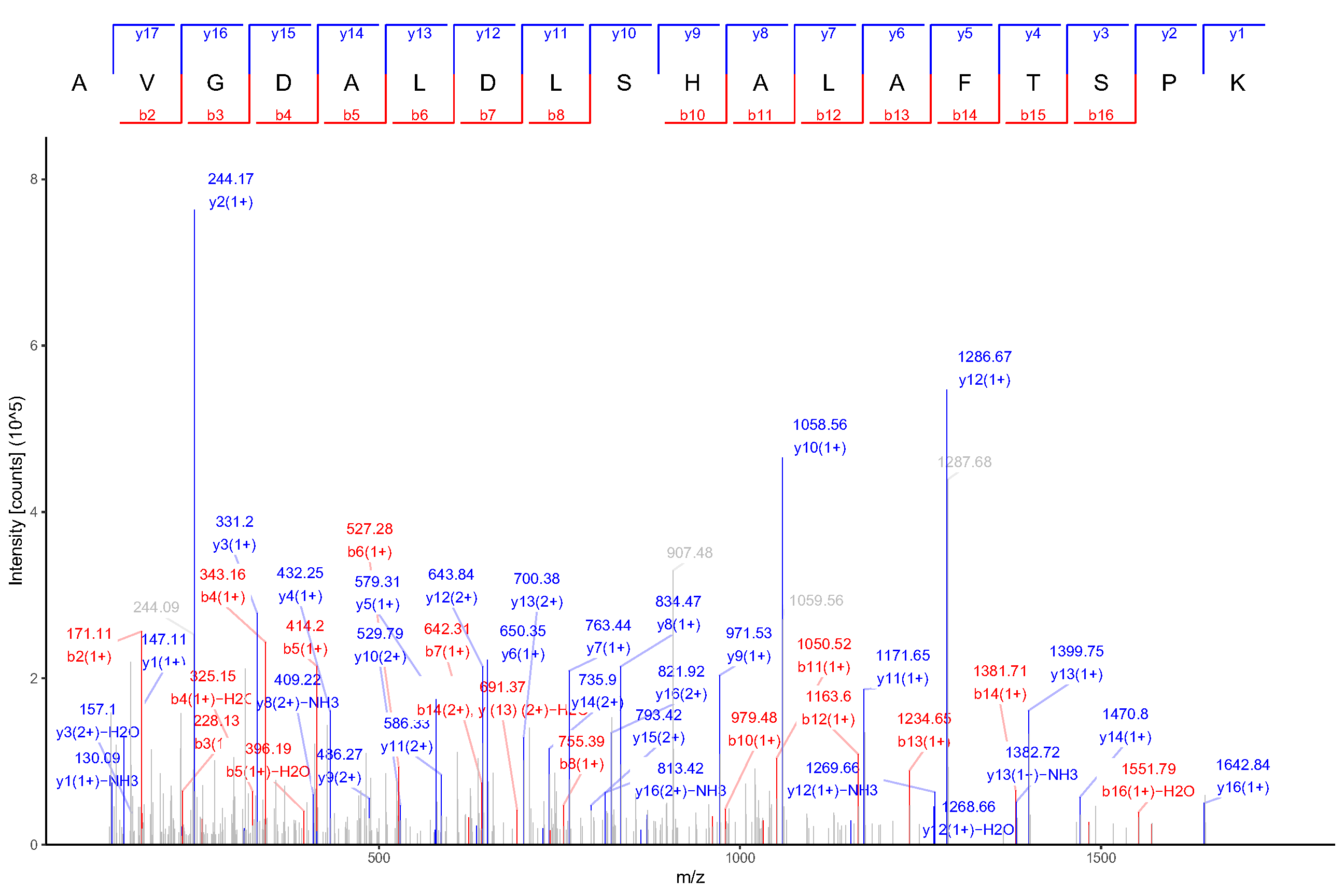

Supplement: S3 Fig — (TIFF) [file ppat.1012147.s008.tiff]

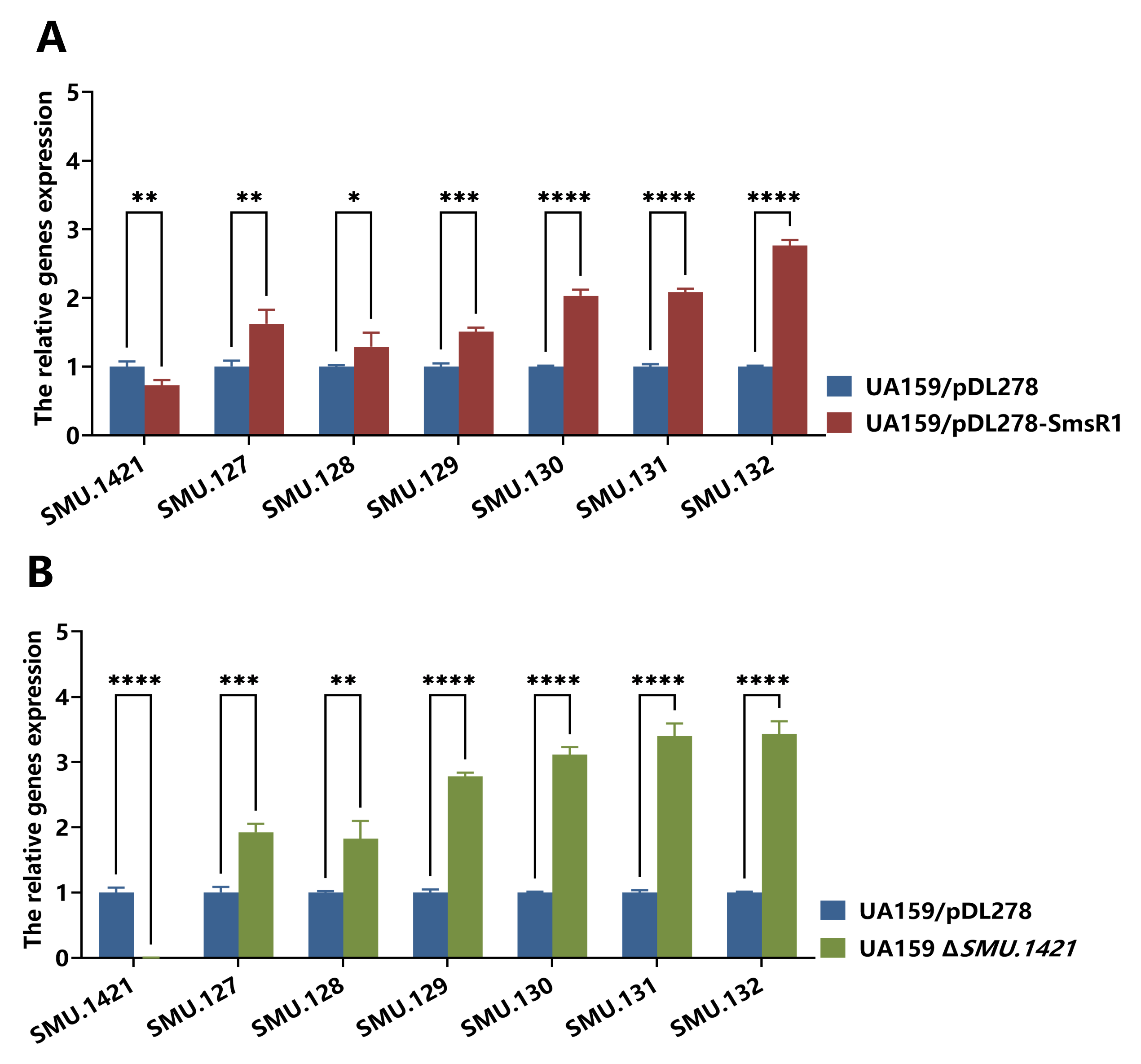

Supplement: S4 Fig — The mRNA levels of SMU.127-SMU.132 in UA159/pDL278, UA159/pDL278-SmsR1 (A) and UA159 ΔSMU.1421 (B) as evaluated by qRT-PCR using the 2 - ΔΔCT method. (TIF) [file ppat.1012147.s009.tif]

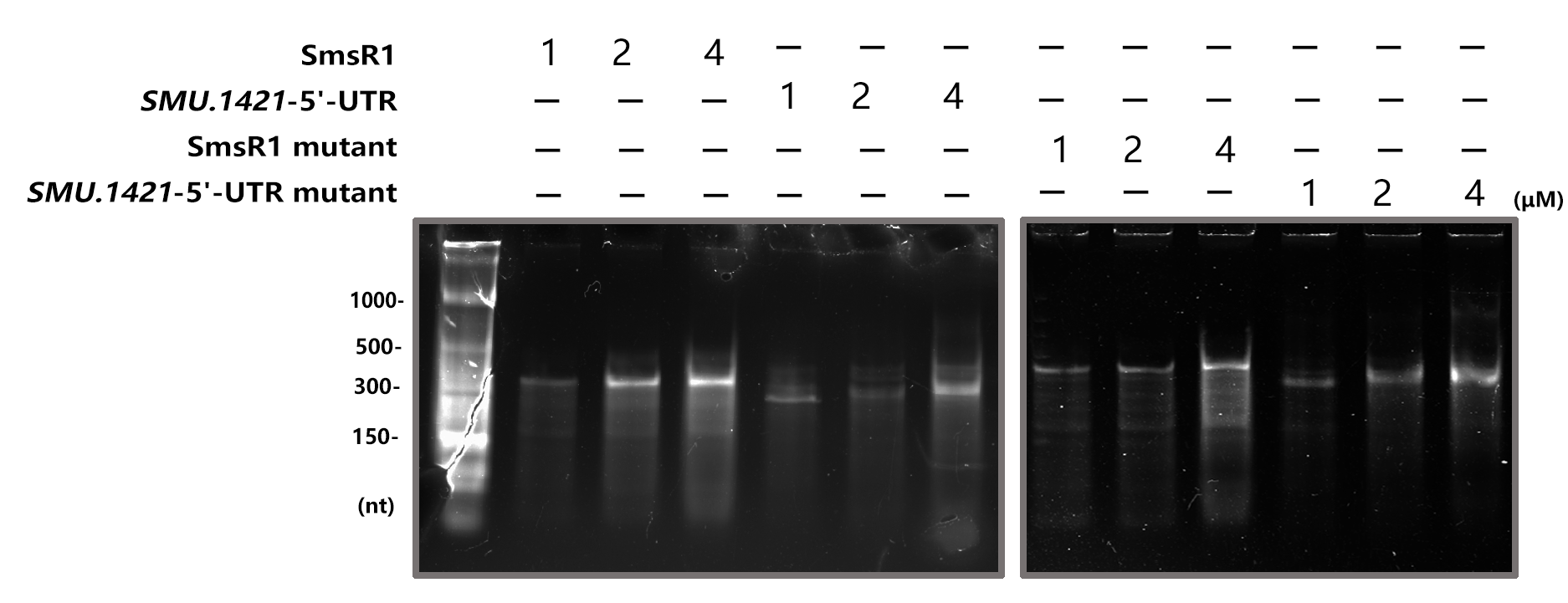

Supplement: S5 Fig — (TIF) [file ppat.1012147.s010.tif]

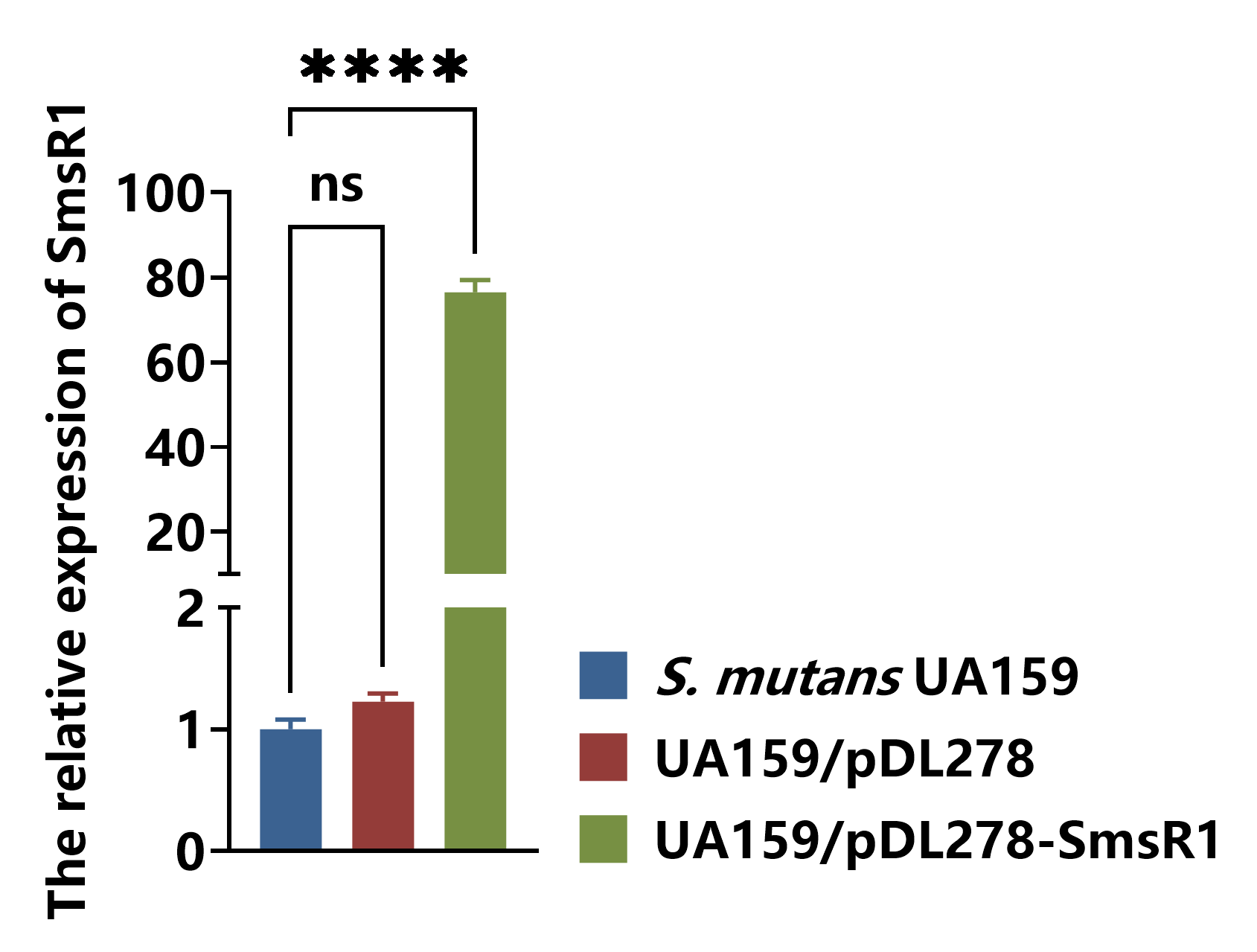

Supplement: S6 Fig — (TIF) [file ppat.1012147.s011.tif]
